# Roles of lncRNAs related to the p53 network in breast cancer progression

**DOI:** 10.3389/fonc.2024.1453807

**Published:** 2024-10-16

**Authors:** Jiarui Song, Qiuxia Cui, Jidong Gao

**Affiliations:** Department of Breast Surgical Oncology, National Cancer Center/National Clinical Research Center for Cancer/Cancer Hospital & Shenzhen Hospital, Chinese Academy of Medical Sciences and Peking Union Medical College, Shenzhen, China

**Keywords:** p53, lncRNAs, breast cancer, gene expression, prognosis

## Abstract

The p53 is a crucial tumor suppressor and transcription factor that participates in apoptosis and senescence. It can be activated upon DNA damage to regulate the expression of a series of genes. Previous studies have demonstrated that some specific lncRNAs are part of the TP53 regulatory network. To enhance our understanding of the relationship between lncRNAs and P53 in cancers, we review the localization, structure, and function of some lncRNAs that are related to the mechanisms of the p53 pathway or serve as p53 transcriptional targets.

## 1 Introduction

**Figure 1:**
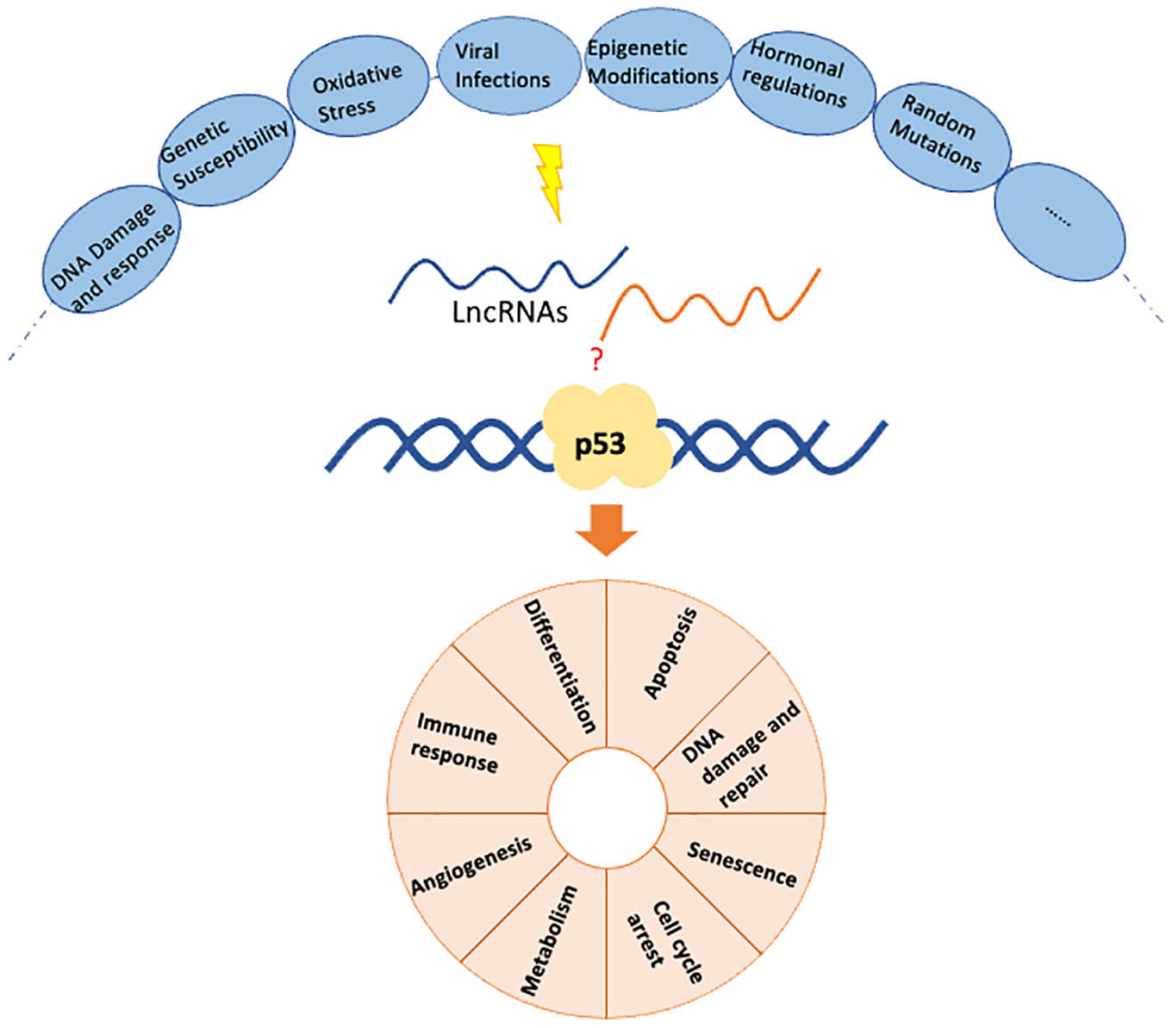
The role of p53 and lncRNAs in regulating breast cancer progression.

Breast cancer is one of the most common malignancies affecting women worldwide (1), characterized by various genetic and epigenetic alterations (2). Among the critical genes involved in breast cancer, TP53, which encodes the tumor suppressor protein p53 (3), is the most commonly altered gene in cancer (4) and is involved in the development of both sporadic and some hereditary breast tumors (3). Non-coding RNAs (ncRNAs) have emerged as critical regulators in the development and progression of breast cancer, among which lncRNAs account for less than 1% (5). However, lncRNAs play critical roles in transcription, post-transcriptional processing, and translation in breast cancer (6). Recent research has uncovered specific role of lncRNAs in gene regulation, interaction with p53, and their potential as therapeutic targets. The tumor suppressor gene p53 and various lncRNAs are significant in regulating breast cancer progression, treatment response, and patient prognosis (**Figure 1**). This study highlights the latest findings in clinical and basic research on p53-associated lncRNAs in regulating breast cancer.

## 2 Long non-coding RNAs and breast cancer

Long non-coding RNAs (lncRNAs) are a diverse group of non-coding RNAs (ncRNAs) longer than 200 nucleotides (7). Instead of evident protein coding capacity, lncRNAs modulate gene expression at various levels, including cellular proliferation, differentiation and development, dosage compensation, chromosomal imprinting, and genomic stability (8), leading to tumor-suppressing or oncogenic functions (9). Statistics from the Human GENCODE exhibit that there are more than 16,000 lncRNAs in the human genome, while others suggest there are exceeding 100,000 human lncRNAs (10, 11). Compared with mRNAs, a higher proportion of lncRNAs are expressed almost only in the nucleus, showing functions related to nuclear processes to control gene expression. lncRNAs can be divided into two categories, cis- or trans-acting, depending on whether the lncRNA impacts nearby genes or performs on long-distance regions (12). Plenty of lncRNAs function in the cytoplasm after being transported from the nucleus, acting as miRNA sponges, regulating mRNA degradation or mRNA translation (13). As significant regulators emerging in cancer biology, lncRNAs also add to the complexity of breast cancer progression (13).

Some lncRNAs can affect the proliferation of breast cancer cells by interacting with key cell cycle regulatory factors. For example, HOTAIR is highly expressed in breast cancer and may promote cell cycle progression by binding to cell cycle-related proteins, leading to excessive proliferation of cancer cells (14, 15). Specific lncRNAs play an important role in the metastasis and invasion of breast cancer cells. They can regulate the epithelial-mesenchymal transition (EMT) process, enabling cancer cells to acquire the ability to migrate and invade. In addition, some lncRNAs can also create favorable conditions for the metastasis of cancer cells by affecting processes such as extracellular matrix remodeling and angiogenesis. Some lncRNAs are closely related to chemotherapy resistance and DNA damage repair in breast cancer. For example, NEAT1 may promote homologous recombination repair or non-homologous end joining repair pathways and affect the response of breast cancer cells to DNA damage caused by radiotherapy and chemotherapy (16).

## 3 Role of p53 in breast cancer

p53 is a key tumor suppressor protein involved in maintaining genomic stability (17). As the major regulatory transcription factor, p53 is reported being activated during the stress response liking replicative stress, oxidative stress, hypoxia, DNA damage (18), nutrient deprivation, and telomere shortening (8). Mutations in the p53 gene are frequent in breast cancer and are associated with a loss of tumor suppressive function or gain of oncogenic properties (19). These mutations contribute to tumor progression, metastasis, and resistance to therapies.

While in breast cancer, the mutation of the p53 gene can be triggered by a variety of factors including: 1) DNA Damage and Repair Deficiencies. Exposure to ultraviolet (UV) radiation, tobacco smoke, chemical carcinogens, and ionizing radiation can cause DNA damage, leading to mutations in the p53 gene (20). Mutations in genes responsible for DNA repair, such as BRCA1 and BRCA2, increase the likelihood of p53 mutations (21). 2) Genetic Susceptibility. Familial breast cancer often involves inherited mutations in BRCA1, BRCA2, and other genes. Li-Fraumeni syndrome, associated with inherited p53 mutations, significantly increases the risk of breast cancer and other cancers (22). 3) Oxidative Stress: Oxidative stress generated by reactive oxygen species (ROS) can cause oxidative DNA damage, leading to mutations (23). 4) Viral Infections: Infections with certain viruses, such as human papillomavirus (HPV), can lead to degradation or inactivation of p53 protein (24). 5) Epigenetic Modifications: Abnormal methylation patterns can lead to genomic instability and increased mutation rates in the p53 gene (25). 6) Hormonal Factors: Estrogen, for instance, promotes the proliferation of breast epithelial cells, potentially increasing the risk of mutations during cell division (26). 7) Random Mutations: Spontaneous DNA Replication Errors accumulate over time, particularly in rapidly dividing cells, contributing to mutations in genes like p53 (27). In breast cancer, mutations in p53 are common and can lead to the loss of its tumor suppressor functions, thereby contributing to cancer progression, resistance to therapy, and poor prognosis. Understanding these processes helps in developing targeted therapies that can restore p53 function or mimic its activity.

Furthermore, p53 is involved in the following biological processes related to breast cancer. 1) induce cell cycle arrest in response to DNA damage (28). 2) promotes apoptosis in cells with irreparable DNA damage (29). 3) enhances the DNA repair process by upregulating genes involved in nucleotide excision repair and base excision repair pathways (30). 4) induce cellular senescence in response to oncogenic stress or extensive DNA damage (31). 5) influences cellular metabolism by regulating genes involved in glycolysis, oxidative phosphorylation, and antioxidant defense (32). 6) suppress angiogenesis, which is essential for tumor growth and metastasis (33). 7) has a role in modulating the immune response against tumor cells (34).

## 4 The direct or indirect regulation between p53 and lncRNAs in breast cancer

**Figure 2:**
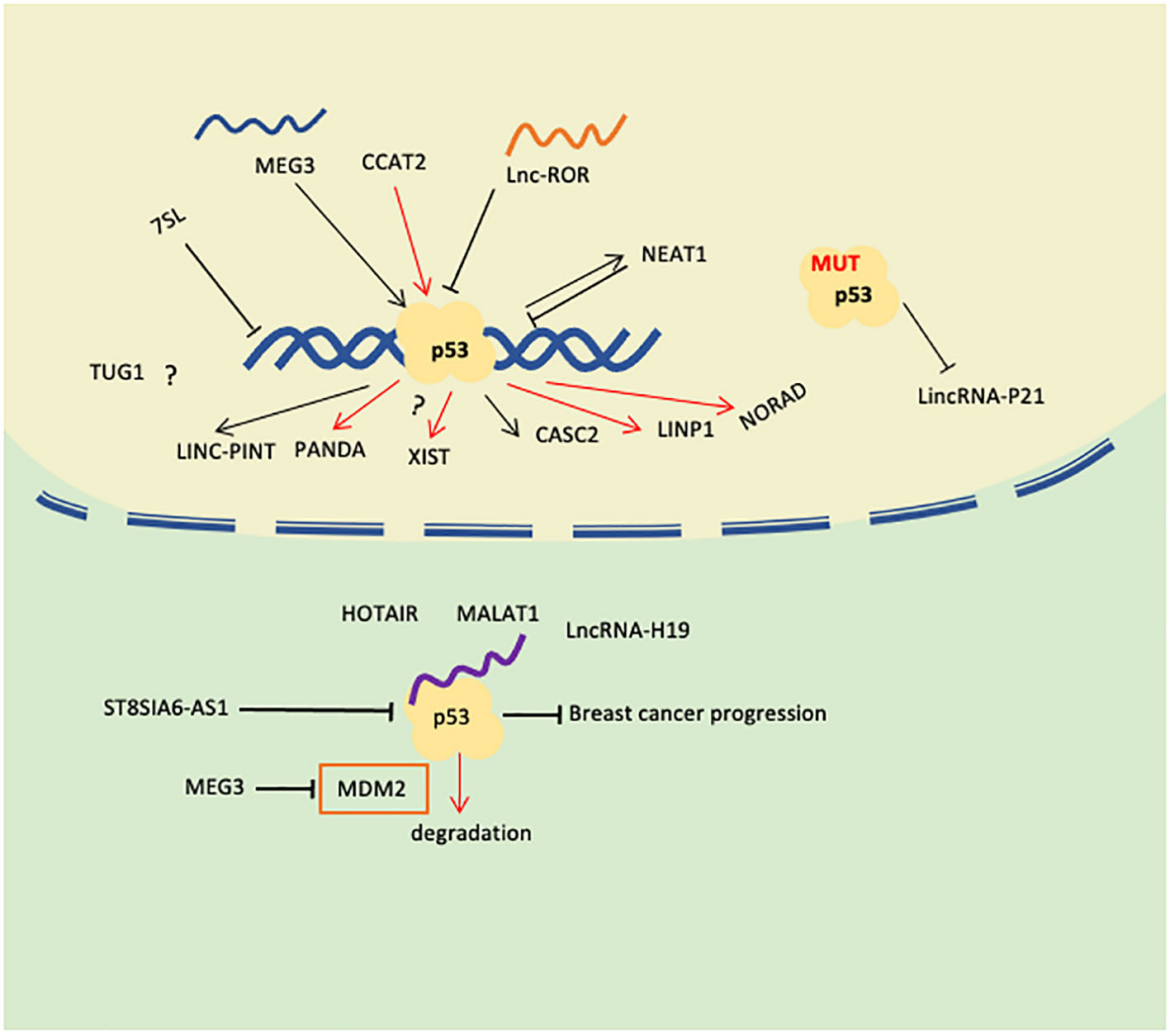
Role of lncRNAs correlated with p53 in Breast Cancer(rows in red mean promoting breast cancer progression or chemotherapy).

p53 can directly or indirectly regulate the expression of some lncRNAs. Some lncRNAs can interact with p53 to regulate the transcriptional activity or protein stability of p53, thereby affecting the regulatory role of p53 on breast cancer cells. p53 and lncRNA may have a synergistic effect in the occurrence and development of breast cancer. For example, mutation or loss of function of p53 may lead to abnormal expression of some lncRNAs, and these lncRNAs further promote the progression of breast cancer. At the same time, abnormal expression of some lncRNAs may also affect the function of p53, thereby aggravating the malignancy of breast cancer. Therefore, both p53 and lncRNA play important roles in breast cancer, and there is a complex association between them. In-depth study of the relationship between lincRNA and p53 in breast cancer can help us uncover new molecular mechanisms, identify potential biomarkers, develop targeted therapies, and improve treatment efficacy. The expression levels or activities of specific lincRNAs and p53 may serve as biomarkers for breast cancer diagnosis, prognosis, or response to treatment. According to the publications reviewed, the relationship between p53 and lncRNAs could be stated from the following aspects (**Figure 2**; **Table 1**). We have searched the recently published works about p53-related lincRNAs from the Pubmed database, mainly using the keywords as breast cancer, p53, and lincRNAs. The following roles are what we were able to access until the paper was submitted.

**Table 1:** lncRNAs related with P53 network in breast cancer.

lncRNA	Functions	subtype	interact with p53	reference
MALAT1	promote migration and invasion	Breast cancer	Depletion of MALAT1 resulting in activation of p53 and its target genes	(35–43)
MEG3	tumor suppressor	TNBC	induces accumulation of p53 by reducing the levels of MDM2 expression, which mediates p53 degration	(44–47)
7SL	upregulated in cancer cells	Breast cancer	The interaction of 7SL with TP53 mRNA reduced p53 translation	(48–50)
HOTAIR	elevated expression correlated with metastasis and death		HOTAIR may affect proliferation, apoptosis, migration and invasion of MCF-7 cells through regulating the P53/Akt/JNK signaling pathway	(14, 15, 51–55)
NEAT1	promoting the progression	Breast cancer	Neat1 ablation slightly decreased DNA damage in PyVT tumors, as determined by phosphorylation of ATM/ATR including p53,	(56–62)
lincRNA-RoR	negative regulator of p53	in er positive breast cancer cells	form an autoregulatory feedback loop : p53 transcriptionally induces RoR expression, and in turn, RoR inhibits p53 translation through hnRNP I.	(63)
ST8SIA6-AS1	promotes cell proliferation and metastasis	TNBC	target miR-145-5p/CDCA3 to inactivate the p53/p21 signaling pathway	(64–66)
CASC2(Cancer Susceptibility Candidate 2)	tumor suppressor	her2 1+ higher than her2 negative	p53 binds to specific response elements in the CASC2 promoter region, activating its transcription.	(67–69)
XIST	high XIST expression had poor outcomes	BRCA1-deficient patients	p53 is required for normal Xist expression and X chromosome inactivation	(70, 71)
PANDA	high levels of PANDA expression had poor prognosis and resistant to chemotherapy		unknow in breast cancer	(69, 72)
LINK-PINT	tumor suppressor	TNBC	p53-induced LINK-PINT transcription	(73, 74)
LINP1	a potential oncogene	TNBC	p53 was identified as a regulator of LINP1	(75, 76)
LincRNA-p21	positively associated with the response to neoadjuvant chemotherapy	ER-positive breast tumor	ERα inhibited lincRNA-p21 expression by redirecting mutp53 to increase tDDB2 transcription, leading to enhanced DNA repair and chemoresistance	(77–82)
LincRNA-p21	lincRNA-p21 knockdown macrophages could mitigate breast cancer progression	TAM	Knocking down lincRNA-p21 led to the polarization of macrophages towards the pro-inflammatory M1 phenotype, which was attributed to MDM2 promoting the proteasome-dependent degradation of p53, consequently activating the NF-κB and STAT3 pathways	(83–85)
lncRNA H19	promote cell proliferation, migration, and invasion, induce cell cycle arrest, and lead to abnormal expression of EMT markers	TNBC	counteract the tumor suppressor p53 and in turn promotes the EMT process	(86)
NORAD	high levels of NORAD expression had poor prognosis	TNBC	is activated in response to DNA damage in a manner dependent on the p53 protein	(87, 88)
(CCAT2)	High CCAT2 expression had lymph node metastasis	ER-positive breast tumor	CCAT2 inhibits p53-induced activation of miR-145, and its overexpression enhances drug resistance	(89, 90)
(TUG1)	promote cell proliferation, cell migration, and invasion	TNBC	low TUG1 levels notably associated with mutant p53 expression and lymph node metastasis	(91–95)

### 4.1 MALAT1

MALAT1 (Metastasis Associated Lung Adenocarcinoma Transcript 1) is a broadly studied lncRNA with a length of ∼8000 nt, which was firstly founded as a prognostic marker in non-small cell lung cancer (35). As a conserved lncRNA, MALAT1 is specially localized in nuclear speckles, a sub-nuclear domain suggested to coordinate RNA polymerase II transcription, pre-mRNA splicing and mRNA export, in a transcription-dependent manner (36–38). Cancer associated MALAT1 regulates cellular proliferation by modulating the expression and/or pre-mRNA processing of cell cycle-regulated transcription factors (39). Associated with hyperproliferation and metastasis, MALAT1 is not only highly expressed in lung cancer (40), but also regulate migration and invasion of breast cancer cells (41). Depletion of MALAT1 leads to cell cycle arrest with prominently reduced cellular proliferation, at the same time, resulting in activation of p53 and its target genes (39). Magdalena Pruszko et al. demonstrated that MALAT1 could be bridged to mutant p53 and ID4 proteins in breast cancer cells (42). The mutant p53 and ID4 delocalize MALAT1 from nuclear speckles and favor its association with chromatin, which enables aberrant recruitment of MALAT1 on VEGFA pre-mRNA. The VEGFA isoforms expression was associated with ID4 expression specifically in basal-like breast cancers carrying p53 mutations (42). Abdel-Latif M et al. showed a repression of MALAT1 in MQG (Methoxylated Quercitin Glycoside) treated MDA-MB-231 (43).

### 4.2 MEG3

MEG3 (Maternally Expressed Gene 3), as an imprinted lncRNA on human chromosome 14q (44), induces accumulation of p53 by reducing the levels of MDM2 expression, which mediates p53 degration (45). But tumor suppression by MEG3 was found to be both p53-dependent and p53-independent (45). MEG3 contains a 356-nucleotide nuclear retention element related to U1 snRNP, which in turn retains MEG3 in the nucleus (46). The overexpression of MEG3 resulted in increased breast cancer cell apoptosis, not only via upregulating the ER-stress and proapoptotic proteins, but by activating the NF-κB and p53 signaling pathways. Pharmacological inhibition of NF-κB completely abolished MEG3 induced activation of p53 (47), making it a potential biomarker and therapeutic target in breast cancer.

### 4.3 7SL

The 300-nt long lncRNAs 7SL (NR 002715; gene name RN7SL1) forms a ribonucleoprotein complex (RNP)with six signal recognition proteins(SRPs) (48) and. was exhibited to be broadly upregulated in several cancer cell types (liver, lung, breast, stomach) (49). 7SL forms a partial hybrid with the 3’-untranslated region (UTR) of p53 mRNA, which encodes the tumor suppressor p53, leading to the reduction of p53 translation (50). On the contrary, silencing 7SL results in enhanced binding of HuR to p53 mRNA, leading to the promotion of p53 translation (50). The competition between 7SL and HuR for binding to p53 3’UTR determined the magnitude of p53 translation, in turn affecting p53 levels and the growth suppressive function of p53 (50). Therefore, targeting 7SL may be effective in the treatment of cancers with reduced p53 levels.

### 4.4 HOTAIR

HOTAIR (HOX Transcript Antisense RNA) is a conserved 2.1kb transcript produced from the HOXC locus on chromosome twelve and is composed of six exons, acting not only as a molecular guide but a scaffold for several chromatin-modifying complexes (51). As one of the most remarkably tumor associated lncRNAs (52), HOT genes are tightly regulated during their development and dysregulated (mostly over-expressed) in different cancer types (15), especially highly induced in breast cancer samples. The elevated HOTAIR expression was correlated with metastasis and death (53). Y. Yu et al. exhibited that knockdown of HOTAIR in MCF-7 cells induced significant increase in the expression of P53, and obvious decrease in the expression of MDM2(Mouse double minute 2 homolog), AKT, JNK on both mRNA and protein level. MDM2 is a negative regulator of p53 (54). Akt and JNK are crucial in the carcinogenesis of breast cancer (55). Therefore, HOTAIR may affect proliferation, apoptosis, migration and invasion of MCF-7 cells through regulating the P53/Akt/JNK signaling pathway (14). That means HOTAIR may have the chance to become a therapeutic target for the treatment of breast cancer.

### 4.5 NEAT1

NEAT1 (Nuclear Enriched Abundant Transcript 1) is a ∼3.7 knt single-exon lncRNA (56). It localizes to paraspeckle sub-organelles and underlies the complex organization and functions of paraspeckles (57), acting as a transcriptional hub for numerous oncogenes (58). NEAT1 could promote the growth and survival cancer cells (59, 60) and chemoresistance in breast cancer cells (61). NEAT1 is a confirmed target gene of p53 (62). p53 regulates NEAT1 expression to promote paraspeckle formation, and in turn, NEAT1 paraspeckles reduce replication-associated DNA damage and p53 activation. This reveals an autoregulatory negative feedback loop that mitigates p53 activity in DNA-damaged cells (16). In addition, NEAT1 is up-regulated by 5-fluorouracil in wild-type p53 breast cancer cells but not in mutant-p53 cells (61). the histone acetylation at the NEAT1 promoter was induced by knocking down of MED12 (Mediator Complex Subunit 12), resulting in elevated NEAT1 mRNA levels and a chemoresistant phenotype. This chemoresistant phenotype can be partially reversed by NEAT1 knockdown in p53 wild-type cells (61). This novel mechanism of chemoresistance dependent on NEAT1 in p53 wild-type breast cancer cells provide a promising target for breast cancer therapies (61).

### 4.6 LincRNA-RoR

The human lincRNA-RoR (Regulator of Reprogramming) has been identified as a powerful negative regulator of p53, through direct interaction with heterogeneous nuclear ribonucleoprotein I (hnRNP I), leading to the inhibition of p53 translation after DNA damage (63). A critical 28-base sequence within RoR, which contains hnRNP I binding motifs, is essential and sufficient for this repression of p53. RoR disrupts p53-mediated cell cycle arrest and apoptosis via an autoregulatory feedback loop where p53 transcriptionally induces RoR expression, and in turn, RoR inhibits p53 translation through hnRNP I. This RoR-hnRNP I-p53 axis may constitute an additional surveillance mechanism for cells in breast cancer (63).

### 4.7 ST8SIA6-AS1

ST8SIA6-AS1, also known as APAL, is an lncRNA that is overexpressed in several cancers and associated with a poor prognosis. Its suppression triggers mitotic catastrophe and substantial apoptosis in human cancer cells (64). It has been found that ST8SIA6-AS1 facilitates breast tumorigenesis by linking up PLK1 and Aurora A to increase PLK1 phosphorylation (64). In triple-negative breast cancer (TNBC) cells, ST8SIA6-AS1 is notably overexpressed. Knockdown of this lncRNA results in decreased cell proliferation, cell cycle arrest, reduced migration and invasion *in vitro*, and inhibited tumor growth *in vivo*. Mechanistically, ST8SIA6-AS1 boosts the expression of CDCA3 (cell division cycle associated protein 3) and inhibits the p53/p21 pathway by targeting miR-145-5p. Through its interaction with miR-145-5p, ST8SIA6-AS1 enhances CDCA3 expression and inactivates p53/p21 signaling, contributing to its oncogenic role in TNBC. Therefore, ST8SIA6-AS1 inactivates p53/p21 signaling to promote breast cancer progression. These insights indicate that ST8SIA6-AS1 could be a promising molecular target for the treatment of TNBC (65, 66).

### 4.8 CASC2

CASC2 (Cancer Susceptibility Candidate 2) has been explored in various cancers, including breast cancer, for its potential tumor-suppressing roles. The expression of CASC2 is triggered by p53 in response to cellular stress or DNA damage. p53 binds to specific response elements in the CASC2 promoter region, activating its transcription. Additionally, CASC2 exerts its tumor-suppressive effects through various mechanisms, interacting with miRNAs, proteins, and other molecular targets to modulate their activity. For instance, CASC2 acts as a competitive endogenous RNA (ceRNA), sponging miRNAs that target tumor suppressor genes, thereby protecting these genes from miRNA-mediated repression. CASC2 also interacts with the protein PTEN, enhancing its tumor-suppressive functions (67). Reduced CASC2 expression in breast cancer is linked to poor prognosis, higher tumor grade, and increased metastatic potential. CASC2 could be a valuable biomarker for breast cancer diagnosis and prognosis. CASC2 interacts with key signaling pathways involved in cancer progression, such as the PI3K/AKT and Wnt/β - catenin pathways (68). Research suggests that CASC2 can sensitize cancer cells to chemotherapeutic agents, making it a promising target for enhancing treatment efficacy The expression levels of p53, MEG3, CASC2, and PANDA were significantly lower in tumor samples compared to non - tumor samples. CASC2 expression was higher in Her2 1+ cases compared to Her2 negative cases (Beta = 1.85, P value = 0.037). Among these genes, CASC2 exhibited the best performance as a biomarker, with an area under the curve value of 0.78, and sensitivity and specificity values of 56.33% and 88.73%, respectively (P value < 0.0001) (69).

### 4.9 XIST

Previous studies have associated high XIST expression and low 53BP1 (p53 binding protein) expression with poor outcomes in systemic therapy and therapy resistance in BRCA1 - deficient mouse tumor models. However, these associations have not yet been evaluated in BRCA1-deficient patients. The expression levels of XIST and 53BP1 were examined to predict the outcome of HD chemotherapy in 28 BRCA1 - like patients. It was reported that high RNA expression of XIST and low protein expression of 53BP1 did not overlap. Patients with either high XIST or low 53BP1 expression had poor outcomes after HD chemotherapy. In contrast, patients with low XIST and high 53BP1 expression showed significant benefits from this regimen in terms of recurrence - free survival, disease-free survival, and overall survival. Therefore, XIST and 53BP1 may serve as predictive biomarkers in BRCA1 - like breast cancer (70). Delbridge et al. provide evidence that p53 is essential for normal Xist expression and X chromosome inactivation. They demonstrate in two models that partial failure of X chromosome inactivation is linked to female - biased neural tube defects (71). However, the relationship between Xist and p53 needs to be further studied.

### 4.10 PANDA

PANDA (P21 associated ncRNA DNA damage activated) is transcribed from a genomic region adjacent to the CDKN1A (p21) gene, a renowned p53 target involved in cell cycle regulation. Upon DNA damage, p53 binds to the promoter region of PANDA, resulting in its transcriptional activation. PANDA has been demonstrated to inhibit apoptosis in cancer cells (osteosarcoma) by sequestering the transcription factor NF-YA, which is implicated in the expression of pro-apoptotic genes (72). In breast cancer, high levels of PANDA expression have been correlated with poor prognosis and increased resistance to chemotherapy PANDA interacts with various proteins and other non-coding RNAs to exert its effects (69). Targeting PANDA or its interacting partners could offer new therapeutic strategies for treating breast cancer treatment, particularly in cases where p53 is functional but the apoptotic pathways are disrupted. The interplay between p53 and PANDA underscores the complexity of p53 signaling in cancer and the significance of non-coding RNAs in modulating these pathways. Understanding the mechanisms by which p53 controls PANDA expression and function can provide valuable insights into breast cancer pathogenesis and open up new avenues for treatment.

### 4.11 LINC-PINT

LINC-PINT (Long intragenic non-coding RNA p53-induced transcript) functions as a tumor suppressor. LINC-PINT is a direct transcriptional target of p53. In response to cellular stress or DNA damage, p53 binds to its putative response element in the promoter region of LINC-PINT, activating its transcription. Reduced expression of LINC-PINT is often associated with poor prognosis and more advanced clinical stages of tumors. Moreover, the specific regulatory mechanisms of LINC-PINT may provide novel targets for therapeutic interventions, potentially leading to more effective cancer treatments (73). In paclitaxel-resistant cells, a significant reduction in the expression of LINC-PINT was observed. When LINC-PINT is ectopically expressed, both paclitaxel-resistant and wild-type triple-negative breast cancer (TNBC) cells showed increased sensitivity to paclitaxel. Mechanistically, LINC-PINT was found to bind to the RNA-binding protein NONO. Overexpressing LINC-PINT leads to the degradation of NONO in a proteasome-dependent manner. Additionally, analysis of patient samples via an online database indicates that LINC-PINT and NONO have antagonistic functions in various types of breast cancer (74). This suggests that the balance between LINC-PINT and NONO may be crucial in determining the outcome of breast cancer.

### 4.12 LINP1

LINP1 is involved in breast cancer cell proliferation, metastasis, and chemoresistance. Through a genetic screening method guided by clinical data, a lncRNA called LINP1 was found to be significantly upregulated in human triple-negative breast cancer. It was revealed that LINP1 plays a role in enhancing the repair of DNA double-strand breaks by acting as a scaffold that connects Ku80 and DNA-PKCs, thereby coordinating the nonhomologous end joining (NHEJ) pathway. LINP1 is controlled by both p53 and epidermal growth factor receptor (EGFR) signaling, and LINP1 overexpression counteracts the metastatic effects of p53. Notably, inhibition of LINP1 enhances the sensitivity of breast cancer cells to radiotherapy (75). Knockdown of LINP1 leads to reduced breast cancer cell growth by inducing G1-phase cell cycle arrest and promoting apoptosis. Additionally, LINP1 facilitates breast cancer cell metastasis and modulates the expression of markers related to epithelial-mesenchymal transition (EMT). LINP1 levels are found to be elevated in cells resistant to doxorubicin and 5-fluorouracil, indicating its role in inducing chemoresistance. Clinical data show that elevated LINP1 levels in tumors are associated with poorer overall survival and disease-free survival in breast cancer patients (76). Therefore, targeting LINP1 could be a promising therapeutic strategy to reduce chemoresistance and improve patient outcomes.

### 4.13 LincRNA-p21

Mutant p53 (mutp53) often loses its ability to bind to p53 response elements (p53REs) and cannot fully trigger apoptosis (77–79). The detailed mechanism has been reported to be correlated with the expression of long intergenic noncoding RNA-p21 (lincRNA-p21), which enhances chemotherapy resistance by targeting its G-quadruplex structure instead of the p53RE on its promoter (80, 81). Surprisingly, estrogen receptor alpha (ERα) inhibits mutp53-mediated lincRNA-p21 expression by redirecting mutp53 to increase the transcription of damaged DNA binding protein 2 (DDB2), leading to enhanced DNA repair and chemoresistance. The levels of lincRNA-p21 are positively associated with the response of breast cancer patients to neoadjuvant chemotherapy and show an inverse relationship with ER status and DDB2 levels. This reveals that the ER status determines how mutp53 functions in promoting chemoresistance as it switches its target gene preference from lincRNA-p21 to DDB2 (82). These results suggest that inducing lincRNA-p21 expression and targeting DDB2 could be effective strategies to enhance the chemosensitivity of breast cancer patients with mutp53.

Besides, a significant upregulation of lincRNA-p21 was revealed in 4T1-educated macrophages. Knocking down lincRNA-p21 leads to the polarization of macrophages towards the pro-inflammatory M1 phenotype in the tumor microenvironment. This effect may be attributed to MDM2 promoting the proteasome-dependent degradation of p53, consequently activating the NF-κB and STAT3 pathways (83, 84). Tumor-associated macrophages (TAMs) with lincRNA-p21 knockdown induce apoptosis in cancer cells and inhibit their migration and invasion. Furthermore, *in vivo* studies showed that adoptive transfer of lincRNA-p21 knockdown macrophages could mitigate breast cancer progression (85). These results highlight lincRNA-p21 as a critical regulator of TAMs in the tumor microenvironment and suggest potential therapeutic targets for tumors characterized by monocyte/macrophage infiltration.

### 4.14 LncRNA H19

LncRNA H19 (Long non-coding RNA H19) is known to play a role in breast cancer tumorigenesis and metastasis by influencing the epithelial-mesenchymal transition (EMT). Elevated expression levels of both lncRNA H19 and TNFAIP8 are observed in breast cancer tissues and cell lines, particularly in TNBC. Knockdown of either lncRNA H19 or TNFAIP8 has been shown to suppress cell proliferation, migration, and invasion, induce cell cycle arrest, and lead to abnormal expression of EMT markers. Mechanistically, lncRNA H19 appears to counteract the tumor suppressor p53, thereby increasing the expression of TNFAIP8, which in turn promotes the EMT process. Additionally, silencing either lncRNA H19 or TNFAIP8 has been found to inhibit tumorigenesis and lymph node metastasis in xenograft mouse models using MDA-MB-231 cells (86). These findings shed light on a new mechanism involving lncRNA H19 in breast cancer tumorigenesis and metastasis, highlighting the H19/p53/TNFAIP8 axis as a potential therapeutic target, especially for TNBC.

### 4.15 NORAD

The human long non-coding RNA NORAD is a newly discovered molecule that is activated in response to DNA damage in a manner dependent on the p53 protein. NORAD expression has been observed in various cancers, including breast cancer (87). Elevated levels of NORAD expression are detected in a series of human epithelial breast cancer cell lines (MDA-MB-231, MDA-MB-436, and MDA-MB-468), which are classified as the most aggressive subtypes known as triple-negative breast cancer. These findings are consistent with previous research indicating that high levels of NORAD expression in basal-like tumors are linked to a poor prognosis. NORAD plays a crucial role in safeguarding genomic stability by interacting with Pumilio proteins, thereby preventing the repression of their target mRNAs (88). Disruption of NORAD function leads to chromosomal instability and aneuploidy (87). Consequently, reducing NORAD levels was found to sensitize triple-negative breast cancer cells to chemotherapy, potentially due to an increased accumulation of genomic abnormalities and a reduced ability to detect DNA damage.

### 4.16 CCAT2

The long noncoding RNA (lncRNA) CCAT2 (colon cancer-associated transcript 2) is found to affect the cell growth, migration, invasion, and drug sensitivity of breast cancer (BC) cells to 5-fluorouracil (5-Fu), involving miR-145 and p53. High CCAT2 expression is associated with lymph node metastasis, positive progesterone receptor, estrogen receptor, and Ki-67 in BC cells. Silencing CCAT2 upregulates miR-145, which is lowly expressed in drug-resistant BC cells (89). p53 is found to bind to the miR-145 promoter region and increase miR-145 expression. The upregulation of miR-145 induced by CCAT2 silencing is reversed by p53-siRNA (90). In conclusion, CCAT2 inhibits p53-induced activation of miR-145, and its overexpression enhances drug resistance in BC cells to 5-Fu.

### 4.17 TUG1

TUG1 (Taurine-upregulated gene 1) is a long non-coding RNA recently linked to the development of various human cancers. TUG1 is demonstrated to be upregulated in breast cancer (91). Knockdown of TUG1 significantly slows down cell proliferation, cell migration, and invasion in breast cancer cell lines MDA-MB-231 and MDA-MB-436. In MDA-MB-231 and BT549, cisplatin-induced cell growth arrest is remarkably augmented by overexpression of TUG1 and was significantly reduced by TUG1 silencing (92). However, in another study, TUG1 expression is significantly decreased in TNBC cell lines compared with normal breast epithelial cell lines and cell lines of other subtypes of breast cancer. Zhang et al. demonstrated that TUG1 is a direct transcriptional target of p53. In lung cancer, p53 binds to its putative response element in the promoter region of TUG1 (93). In breast cancer, TUG1 expression is significantly reduced in breast cancer tissues and cell lines compared to normal controls, and low TUG1 levels are notably associated with mutant p53 expression and lymph node metastasis (94). *In vitro*, overexpressing TUG1 markedly inhibits cell proliferation by inducing cell cycle arrest and apoptosis in breast cancer cells, while silencing TUG1 leads to increased cell growth by promoting cell cycle progression and altering the expression of cyclinD1 and CDK4. Further functional tests demonstrated that TUG1 overexpression significantly enhances cell migration and invasion, whereas TUG1 knockdown has the opposite effects (95). The mechanism should be further investigated in breast cancer.

## 5 Suggestions for clinical research translation

The study of p53-related lncRNAs regulation is of great significance for clinical breast cancer and can be translated into clinical applications as follows:

### 5.1 Diagnostic significance

P53-related RNAs, such as specific lncRNAs, can serve as potential biomarkers for breast cancer. The abnormal expression levels of these RNAs in breast cancer tissues compared to normal tissues can provide important clues for early diagnosis. For example, detecting the expression of lncRNAs like MEG3, CASC2, and others can help clinicians identify breast cancer at an early stage and improve diagnostic accuracy. Non-invasive or minimally invasive diagnostic methods can be developed by detecting these RNAs in blood or other body fluids, increasing patient acceptance.

### 5.2 Prognostic value

The expression patterns of p53-related RNAs can predict the prognosis of breast cancer patients. For instance, certain lncRNAs may be associated with poor prognosis, higher tumor grade, and increased metastatic potential. This information can help clinicians make more informed decisions about treatment options and patient management.

### 5.3 Therapeutic implications

Targeting p53-related RNAs can provide new therapeutic strategies. For example, inhibiting oncogenic lncRNAs or restoring the function of tumor suppressor lncRNAs can inhibit the growth, metastasis, and drug resistance of breast cancer cells. Understanding the role of p53-related RNAs in chemotherapy resistance can help develop strategies to overcome resistance and improve treatment efficacy. For instance, by targeting specific lncRNAs that are abnormally expressed in drug-resistant cells, the sensitivity of breast cancer cells to chemotherapy drugs can be enhanced.

The knowledge gained from studying p53-related RNA regulation can lead to the development of novel targeted therapies. By understanding the specific functions and interactions of these RNAs, researchers can design drugs or therapeutic strategies that specifically target these molecules to inhibit breast cancer progression. Combining traditional treatment methods with RNA-targeted therapies based on p53 regulation can potentially improve treatment outcomes and reduce the risk of recurrence and metastasis.

## 6 Limitations and future directions

Despite significant progress, several challenges remain in the study of p53-related lncRNAs in breast cancer. These include the functional characterization of lncRNAs, elucidation of their precise mechanisms of action, and development of effective therapeutic strategies targeting these molecules. Developing efficient delivery systems for lncRNA-based therapies to ensure specificity and minimize off-target effects is a major hurdle. Additionally, large-scale clinical trials are needed to validate the prognostic and therapeutic potential of lncRNAs. Future research should focus on addressing these challenges to harness the full potential of p53-related lncRNAs in breast cancer management. Exploring the interaction networks of lncRNAs, miRNAs, and mRNAs could provide a comprehensive understanding of their roles in cancer biology. Continued research in this field is likely to lead to breakthroughs in personalized medicine, offering new hope for patients with advanced and resistant forms of breast cancer.

## 7 Conclusion

Breast cancer is a major global health challenge. Understanding its molecular mechanisms, classification, and treatment options is crucial. P53-related long non-coding RNAs are a hot research field in breast cancer biology. By elucidating the interaction between p53 and lncRNAs, it is expected to reveal new diagnostic markers and therapeutic targets and improve patient prognosis. Resolving existing controversies, optimizing screening and treatment strategies, and developing new targeted inhibitors are essential for reducing the burden of breast cancer worldwide.
